# The Unravelling of the Genetic Architecture of Plasminogen Deficiency and its Relation to Thrombotic Disease

**DOI:** 10.1038/srep39255

**Published:** 2016-12-15

**Authors:** Laura Martin-Fernandez, Pascual Marco, Irene Corrales, Raquel Pérez, Lorena Ramírez, Sonia López, Francisco Vidal, José Manuel Soria

**Affiliations:** 1 Unit of Genomics of Complex Diseases, Biomedical Research Institute Sant Pau (IIB-Sant Pau), Barcelona, Spain; 2 Department of Haematology, Hospital General Universitario, Alicante, Spain; 3 Congenital Coagulopathies, Blood and Tissue Bank, Barcelona, Spain; 4 Molecular Diagnosis and Therapy, Vall d’Hebron Research Institute, Universitat Autònoma de Barcelona (VHIR-UAB), Barcelona, Spain

## Abstract

Although plasminogen is a key protein in fibrinolysis and several mutations in the plasminogen gene (*PLG*) have been identified that result in plasminogen deficiency, there are conflicting reports to associate it with the risk of thrombosis. Our aim was to unravel the genetic architecture of *PLG* in families with plasminogen deficiency and its relationship with spontaneous thrombotic events in these families. A total of 13 individuals from 4 families were recruited. Their genetic risk profile of thromboembolism was characterized using the Thrombo inCode kit. Only one family presented genetic risk of thromboembolism (homozygous carrier of *F12* rs1801020 and *F13A1* rs5985). The whole *PLG* was tested using Next Generation Sequencing (NGS) and 5 putative pathogenic mutations were found (after *in silico* predictions) and associated with plasminogen deficiency. Although we can not find genetic risk factors of thrombosis in 3 of 4 families, even the mutations associated with plasminogen deficiency do not cosegregated with thrombosis, we can not exclude plasminogen deficiency as a susceptibility risk factor for thrombosis, since thrombosis is a multifactorial and complex disease where unknown genetic risk factors, in addition to plasminogen deficiency, within these families may explain the thrombotic tendency.

The plasminogen protein plays a pivotal role in fibrinolysis and wound healing. Briefly, this protein generates the active enzyme plasmin by tissue-type plasminogen activator (t-PA) or urokinase-type plasminogen activator (u-PA). Plasmin, which is a serine protease enzyme, is essential for the dissolution of blood clots in a fibrin-dependent manner. In addition, plasmin has different substrate specificities and functions that are important in wound healing, such as other matrix glycoproteins or the activation of matrix metalloproteinases^1^. The plasminogen protein is encoded by the PLG gene, which is located on Chromosome 6q26, has 19 exons and is 51,861 bp in length (http://genome.ucsc.edu/; February 2009 release of the human genome, GRCh37/hg19)^2^. Numerous mutations have been described in the *PLG* locus that cause plasminogen deficiency, such as missense, nonsense, frameshift, splice site, deletion and insertion mutations^3^,^4^.

Plasminogen deficiency is a rare disorder that has been classified as type I or hypoplasminogenemia, described first by Hasegawa *et al*. in ref. 5, and type II or dysplasminogenemia, which was described for the first time by Aoki *et al*. in ref. 6. In type I, both plasminogen activity and antigen levels are decreased. In contrast, type II is characterized by decreased plasminogen activity but normal antigen levels^4^,^7^. Both functional and antigen assays have been used in clinical diagnosis. Molecular genetic analysis has supported the clinical diagnosis, and identified individuals at risk for plasminogen deficiency. It may be used also for prenatal diagnosis^8^. Currently, different approaches have been reported for testing for genetic mutations: single-strand conformation polymorphism analysis of PCR products encompassing exons and intron boundaries, restriction fragment length polymorphism technique and Sanger sequencing of both PCR amplicons of exons and flanking intronic regions and long range (LR) PCR amplicons^4^,^9^,^10^.

Plasminogen deficiency results in a reduction in the degradation of fibrin and thus affects wound healing. For example, ligneous conjunctivitis, congenital occlusive hydrocephalus and juvenile colloid milium have been reported^1^,^3^. Among these, ligneous conjunctivitis is the most common and is characterized by chronic tearing, erythema of the conjunctiva and white, yellow-white, or red thick masses with a wood-like consistency. This chronic conjunctivitis is associated with homozygous or compound heterozygous type I patients. Of note, heterozygous type I and type II patients are asymptomatic^3^. Some studies^6^,^11^ suggested that there was an association between plasminogen deficiency and thrombosis due to impaired fibrinolysis. However, these studies involved few patients of sympthomatic thrombophilia. In addition, most family studies showed that only the probands had thrombosis. It is noteworthy that some risk factors of thrombosis such as activated protein C (APC) resistance were unknown when this association was reported^12^. More recent reports supports the hypothesis that this disorder by itself is not a risk factor of venous or arterial thrombosis^3^,^12^,^13^. Also, it is suggested that the diminished plasminogen capability of fibrinolysis may be compensated by the action of alternative enzymes in the blood^3^.

In our study, we identified 4 Spanish patients that had suffered spontaneous thrombotic events. We identified a plasminogen deficiency in these patients and in several of their relatives. Our aims were to characterize the genetic risk factors of thromboembolism in these families and to detect the genetic mutations that were involved in the plasminogen deficiency. We used a new method to diagnose the deficiency based on Next Generation Sequencing (NGS) of *PLG*. We evaluated also the relation of these causal genetic mutations to the thrombotic outcomes to elucidate the role of plasminogen deficiency in thrombotic disease.

## Material and Methods

### Subjects

A total of 13 individuals from 4 Spanish families (A, B, C and D) were recruited at the Hospital General Universitario de Alicante. These families were selected through probands with a positive history of spontaneous thrombosis and plasminogen deficiency. The latter was defined as a functional activity below 72% (normal range, 72% to 127%). The characterization of these families is presented in Table 1. Clinical manifestations related to plasminogen deficiency were not identified and no classification of type I or type II was performed. All of the probands and one relative had experienced a spontaneous deep venous thrombosis of the legs or an arterial thrombotic event. The number of individuals per family varied from 1 to 5 and they ranged in age from 13 to 74 years. A total of 9 individuals were selected from the 4 families for genetic analysis, which included the probands and individuals with the lowest functional plasminogen deficiency values. At least 2 relatives of every family were selected for inclusion, except for one family with no relatives.

The study is part of the clinical routine of the Hospital General Universitario de Alicante for thrombophilia patients conducted in accordance with the Good Practice Guidelines that included an informed consent from all individuals prior to inclusion. The studies were conducted in accordance with the Declaration of Helsinki.

### Blood Collection and Phenotype Determinations

Blood samples were obtained from the antecubital vein and anticoagulated with 3.8% sodium citrate in the proportion 1:10. The blood samples were centrifuged for 10 minutes at 3,500 g within 15 minutes after collection. The poor-platelet plasma (PPP) samples were immediately frozen and stored at −80 °C until tested. DNA was extracted from whole blood collected in EDTA using a standard salting out procedure^14^. The functional plasminogen activity was performed in PPP using a chromogenic substrate assay, activated by tissue plasminogen activator (Instrumentation Laboratory, Werfen Group, MA, USA).

### Genetic Profile of Thromboembolism

The Thrombo inCode (TiC) kit (Ferrer in Code, Barcelona, Spain) was used to determine the genetic profile of thrombosis. The TiC kit identified the alleles of 12 genetic variants: *F5* (rs6025, rs118203906, rs118203905), *F2* rs1799963, *F12* rs1801020, *F13A1* rs5985, *SERPINC1* rs121909548, *SERPINA10* rs2232698 and the A1 blood group (rs8176719, rs7853989, rs8176743 and rs8176750). This genetic profile has been validated and reported in the literature^15^. The TiC kit was applied by Taqman assays run in an ABI 7500 instrument according to manufacturer’s instructions.

### Primer Design and PCR Amplification

A total of 55,184 bp of the *PLG* locus (GRCh37/hg19 chr6:161,123,225-161,175,085; NM_000301.3) were amplified using 7 LR-PCR amplicons. The primer sequences, positions and PCR amplicon size are shown in Table 2. The PCR primers were designed to specifically amplify the *PLG* and to avoid co-amplifications in view of the high homology between *PLG, LPA* family genes, pseudogenes and plasminogen-like genes^10^. The overlapped PCR amplicons covered all of the exons, introns, 5′-UTR, 3′-UTR and approximately 1,500 bp of the 5′-promoter region. The PCR amplicons designed were tested for target specificity by Sanger sequencing as described below.

LR-PCR amplicons were generated using the SequalPrep Long PCR Kit with dNTPs (Invitrogen, Thermo Fisher Scientific Inc., MA, USA). The LR-PCR mix solution contained ~50 ng of DNA, SequalPrep 1X reaction buffer, 0.4 μl of dimethylsulfoxide (DMSO), SequalPrep 1X enhancer B, 0.75 μM of forward and reverse primers and 1.8 units of SequalPrep Long Polymerase in a total volume of 20 μl. After initial denaturation at 94 °C for 2 minutes (min), 10 cycles of 94 °C for 10 seconds (sec), 64 °C for 30 sec, and 68 °C for 18 min were performed, followed by 22 cycles of 94 °C for 10 sec, 64 °C for 30 sec, and 68 °C for 18 min (+20 sec/cycle). In addition, an elongation step at 72 °C for 5 min was performed.

Every PCR amplicon was run on 0.7% agarose gel electrophoresis and visualized using SYBR safe (Invitrogen, Thermo Fisher Scientific Inc.). PCR amplicons were quantified by the Qubit technology (Invitrogen, Thermo Fisher Scientific Inc.) and a normalized pool of the 7 PCR amplicons was prepared for each individual by mixing equimolar amounts. Finally, the PCR pools were adjusted at 0.2 ng/μl for preparing the libraries.

### Library Preparation and NGS

The sequencing libraries were prepared from pooled PCR amplicons using the Nextera XT DNA Sample Preparation kit (Illumina, San Diego, CA, USA) with double indexing, following the standard manufacturer’s protocol. We obtained 9 paired-end libraries that were pooled and run simultaneously on an Illumina Miseq sequencing system (Illumina) by the Miseq sequencing reagent kit v2 of 300 cycles (2 × 150 bp paired-end) (Illumina).

### Bioinformatic Analysis

Indexed sequences were de-multiplexed and analyzed individually. The NGS pipeline output, paired sequence files (fastq files format), was used as input for the analysis with the CLC Genomic Workbench (v.6.5) software (CLC Bio - Qiagen, Aarhus, Denmark). The raw data were trimmed with length (minimum 25 bp; maximum 500 bp), ambiguous nucleotide (maximum 2) and quality score (0.05) filters. CLC Genomic Workbench software permitted the alignment of the trimmed reads against the human genome reference (hg19) and *in silico* analysis. Read mapping was performed with specific parameter setting (mismatch count, 2; indel count, 3; length fraction, 0.7; similarity fraction, 0.9). Indels and structural variants tool was used to identify insertions and deletions, inversions, translocations and tandem duplications, applying standard settings. Moreover, adjusted parameters for quality-based variant detection were as follows: minimum coverage, 30x; minimum variant frequency, 25%. Quality-based variant detection results in variant call format (VCF) file were used as input for the Illumina VariantStudio Data Analysis (v.2.1) Software (Illumina) to annotate variants.

### Screening Putative Pathogenic Mutations

For structural variants, we used the following filter parameters to detect pathogenic variations: a) minimum variant ratio, 0.25, b) minimum mapping scores fraction, 0.6, c) consider intronic structural variants located <30 bp from exon flanking boundaries, d) allele frequency from our own NGS variants database of 110 individuals, ≤5%, and e) co-segregation in the family.

The following criteria were applied to identify putative pathogenic mutations within single nucleotide variants (SNV) and small insertions and deletions: a) whether the variant was rare: allele frequency ≤1% from 1000 Genomes (April 2012 v.3)^16^ and NHLBI Exome Variant Server (June 2013 ESP6500SI-V2), b) location referring to Variant Effect Predictor (v.2.8) database: intronic mutations located <30 bp into flanking intronic sequence of each exon were included, c) allele frequency from our own NGS variants database of 110 individuals, ≤5%, in the case of variants not annotated in 1000 Genomes (April 2012 v.3)^16^, and d) co-segregation in the family.

We defined as “new” those putative pathogenic mutations that were not reported in the literature related to the plasminogen deficiency disorder. The amino acid numbering and the nomenclature used for the description of the sequence variations follows the international recommendations of the Human Genome Variation Society (HGVS; http://www.HGVS.org).

### Sanger Validations

Putative pathogenic mutations were validated by Sanger sequencing^17^ in probands and family members. The sequences were mapped against the *PLG* locus (GRCh37/hg19 chr6:161,123,225-161,175,085; NM_000301.3) using the CLC Genomic Workbench (v.6.5) software.

### *In Silico* Analyses

The *in silico* prediction that evaluate functional effects of putative pathogenic mutations was performed using the Alamut Visual (v.2.6.1) software (Interactive Biosoftware, Rouen, France). Changes in splicing sites were predicted using NNSplice and Human Splicing Finder tools. For splicing variants interpretation, a splicing site change was considered as potentially deleterious when a variation between the native and the mutation score of more than 10% was observed in both algorithms^18^. Missense prediction tools included SIFT, PolyPhen-2, Align GVGD and Mutation Taster. Evolutionary conservation scores were obtained using phyloP and Grantham distances. Interpretation of predictive structural effects of new missense mutations was investigated by using the Project HOPE software^19^.

## Results

### Genetic profile of thromboembolism

We characterized the alleles of 12 genetic variants located within *F2, F5, F12, F13A1, ABO, SERPINA10* and *SERPINC1* loci, included in the TiC kit. Based on these results, all of the individuals were reported as not at genetic risk of thrombotic disease except for the proband of Family D (Table 3). This individual was homozygous for the alleles c.-4T in the *F12* (rs1801020), also known as 46 T risk allele^15^,^20^, and c.103 G>G of the *F13A1* (rs5985), also known as Val34 risk allele^15^.

### NGS results

We amplified 55,184 bp encompassing the *PLG* locus using LR-PCR from 9 individuals. These LR-PCR amplicons were analysed by NGS. Briefly, the percentage of the mapped positions with a depth of coverage above 30x was 95% and the median coverage per individual was 212x. In total, 237 potential structural variants and 301 unique SNV and small insertions and deletions were called. Among the latter, the percentage of indels and exonic variants was 12.6% (38) and 3.7% (11), respectively, and the 55.5% (167) had an allele frequency ≤1% from all population of 1000 Genomes (April 2012 v.3) and from four populations of 1000 Genomes (American, East Asian, African and European). In addition, the 47.8% (144) of the variants had not been reported in dbSNP (v. 137) and 143 out of 144 were within the introns.

### Putative pathogenic mutations

We identified 5 putative pathogenic mutations, including 3 missense variations and 2 potential splicing site mutations (1 synonymous and 1 intronic variants) reported in Table 3. Within the family members, the 5 candidate mutations were confirmed by Sanger sequencing if genomic DNA was available. Also, we performed *in silico* predictions (Table 4). Specifically, any structural variant passed through the filtering criteria for putative pathogenic structural variant detection.

In Family A, which was composed of 3 members, the proband was compound heterozygous in trans for p.Lys38Glu (in exon 2) and p.Gly712Arg (in exon 18) missense variations in *PLG*. This individual had a functional plasminogen activity level of 24%. The variant p.Lys38Glu was also heterozygous in the son with a level of functional plasminogen activity of 67% while another son who was heterozygous for the p.Gly712Arg variation had a functional plasminogen activity of 58%. Of note, both missense variations have been described previously^1^,^3^,^21^ in association with plasminogen deficiency.

The p.Lys38Glu variation was identified also in Family B. This missense mutation was detected as heterozygous in 4 of the 5 members. These 4 individuals included the proband with a functional plasminogen activity level of 47% and 3 family members with functional plasminogen activity level of 58%, 50% and 67%. In contrast, the non-carrier family member showed a normal level of functional plasminogen activity (82%).

In Family C, the new putative pathogenic variation p.Arg261Cys (in exon 7) was heterozygous in 3 of the family members. The functional plasminogen activity was low in these individuals, who included the proband (68%), his sibling (64%) and his mother (64%), in comparison to the level detected in the maternal aunt of the proband, who was a non-carrier family member (101%). The effects of this missense mutation predicted by SIFT, Polyphen-2, Align GDGV and Mutation Taster were deleterious, probably damaging, most likely interfering with protein function and disease causing, respectively. In addition, it was reported as a highly conserved nucleotide and there were large physicochemical differences between Arg and Cys. Project HOPE software revealed differences between the native and the mutant residues in size and charge. The cysteine residue was smaller than the native arginine residue and the positive charge of the arginine was lost as a result of this mutation. Furthermore, the mutated cysteine was located very close to a residue that makes a cysteine bond, and despite this bond which itself is not mutated, it could be affected by the mutation located in its vicinity. The missense variation p.Arg261Cys was characterized as part of a Kringle domain with hydrolase activity, which is essential for the activity of the protein and involved in protein-protein interactions.

The last proband (Family D) had a functional plasminogen activity of 55%. The mutational analysis showed that this individual was compound heterozygous for two potential splice site mutations. Specifically, the p.Lys4Lys synonymous variant in exon 1, which was located in the signal peptide, and the c.1878-6 T>C variant in intron 15. To investigate *in silico* functional consequences of these new putative pathogenic mutations predictive changes in splicing sites were analyzed. By doing so, no alterations at the donor splice site of exon 1 or at the natural acceptor splice site of exon 16 were detected for the synonymous and the intronic variants, respectively.

## Discussion

We present the molecular characterization of 13 individuals from 4 plasminogen deficiency families, in which the probands had suffered a thrombotic event. Since there is doubt as to whether plasminogen deficiency is a risk factor of thrombotic disease by itself or in combination with other abnormalities^1^, we genotyped 12 genetic risk factors of thrombosis coded by *F2, F5, F12, F13A1, ABO, SERPINA10* and *SERPINC1* loci to evaluate the putative role of plasminogen deficiency phenotype in thrombotic disease. We identified a genetic risk profile of thromboembolism in Family D. The proband was homozygous for the risk alleles in *F12* (rs1801020) and *F13* (rs5985). Interestingly, this individual had positive antiphospholipid antibodies also that might have acted as a risk factor in her ischemic stroke. In contrast, Families A, B and C did not show genetic risk profiles of thromboembolism despite suffering thrombotic episodes. Thus, plasminogen deficiency could not be ruled out as an additional risk factor. It is noteworthy that thromboembolism is a common disease with more than 60% of the variation due to genetic risk factors^22^,^23^. However, genetic scores explain only 15% of the variance^15^, so there is still a “missing heritability” that might have hampered the discrimination of plasminogen deficiency as an additional risk factor of thrombotic disease. Also, whether individuals of these families other than the probands will suffer a thrombotic event in the future is not known.

We sequenced the whole *PLG* locus with good coverage of high quality reads to identify the genetic variants that might be involved in the functional plasminogen deficiency of these families using the NGS methodology. Recent advances in NGS have provided high-throughput, economical, sensitive and faster sequencing methodologies^24^,^25^. Because of these advantages, and the fact that many samples can be analyzed simultaneously, NGS is ideal for targeted diagnostic sequencing of monogenic diseases where genetic heterogeneity is expected. New pathogenic mutations have been discovered using NGS^26^,^27^ but to our knowledge, no publications have explored the genetic basis of plasminogen deficiency using NGS. We performed whole gene sequencing to exhaustively examine not only exons but also non-coding regions (promoter, introns, UTR) that might be involved in regulatory functions^28^,^29^,^30^. In addition, LR-PCR provided a fast and cost-effective technique for avoiding the amplification of plasminogen pseudogenes^31^,^32^. Also, LR-PCR in combination with paired-end sequencing allowed us to use algorithms to accurately detect structural variants and avoid the use of additional analyses as MLPA (multiplex ligation-dependent probe amplification) or MAPH (multiplex amplifiable probe hybridization).

We have identified 5 putative pathogenic mutations, which are in concordance with the levels of plasminogen functional activity by intrafamilial analyses. In particular, the mutation p.Lys38Glu (K19E, old nomenclature) has been described^1^,^3^,^21^ widely as the most common molecular genetic defect in association with type I. This mutation has been identified in homozygous, compound heterozygous and heterozygous state^4^ but it is not known why this mutation and others in the *PLG* leads to diverse clinical conditions^8^. In addition, the missense variation p.Gly712Arg (G693R, old nomenclature) has been described in heterozygous state related to type II plasminogen deficiency^21^. Interestingly, we have identified both the p.Lys38Glu and p.Gly712Arg variants as compound heterozygous in one individual. In contrast, for the first time to our knowledge the p.Arg261Cys variation is described in association with plasminogen deficiency. This missense variation was classified as deleterious using *in silico* predictions. Several structural effects in the plasminogen protein have been suggested by Project HOPE. Specifically, changes in size and charge between the native and the mutated residue were predicted, which could cause loss of protein-protein interactions. In addition, Project HOPE software predicted that p.Arg261Cys mutation could disturb the interaction between domains, which might affect the protein’s function also. This variant has been listed previously as variant 6:161137789 C / T in the Exome Aggregation Consortium (ExAC) Browser (Cambridge, MA, http://exac.broadinstitute.org) with an allele frequency of 5.771 × 10^−5^. It is noteworthy that another variation in the same amino acid (c.782 G>A, p.Arg261His, rs4252187) has been annotated in NCBI dbSNP (v.146, http://www.ncbi.nlm.nih.gov/SNP/). Also, we identified the synonymous p.Lys4Lys and the intronic c.1878-6 T>C variation in one individual as new putative pathogenic mutations in association with plasminogen deficiency. Both genetic variations were not predicted by *in silico* analyses to change the splice site natural junction. Despite this, synonymous and intronic variations could cause alterations in protein expression and function^33^,^34^. Thus, further analyses are needed to determine the functional characterization of these mutations.

It is important to note that we identified the whole spectrum of genetic variability of the *PLG* structural gene in these families. However, we did not observe a clear association between the genotype, the number or the type of putative pathogenic mutations in *PLG* with the thrombotic phenotype of these individuals. Moreover, we can not find genetic risk factors of thrombosis in 3 of 4 families. These observations provide us with a scenario where we can not confirm neither exclude plasminogen deficiency as a susceptibility risk factor for thrombosis.

Our view is that thrombosis is a multifactorial and complex disease where several genetic risk factors with environmental situations increase the susceptibility to develop a thromboembolic event. Therefore, in these families unknown genetic risk factors, in addition to plasminogen deficiency, may explain the thrombotic tendency in concrete clinical situations. Thus, we believe that it may be useful the evaluation of plasminogen deficiency in individuals that had suffered a spontaneous thrombotic event and had a negative routing thrombophilia test.

It is important to emphasize that, from a clinical point of view, we detected putative pathogenic mutations that explain the plasminogen deficiency. In these sense, our NGS approach clearly contributed to the genetic knowledge of plasminogen deficiency. We believe that NGS has the potential to identify and characterize the molecular basis of a wide variety of disorders in addition to plasminogen deficiency.

## Additional Information

**How to cite this article**: Martin-Fernandez, L. *et al*. The Unravelling of the Genetic Architecture of Plasminogen Deficiency and its Relation to Thrombotic Disease. *Sci. Rep.* **6**, 39255; doi: 10.1038/srep39255 (2016).

**Publisher’s note:** Springer Nature remains neutral with regard to jurisdictional claims in published maps and institutional affiliations.

## Figures and Tables

**Table 1:** Phenotypic data of the four families with plasminogen deficiency.

Family	Relationship to proband	Age (years)	Age at first episode of thrombosis (years)	Sex	Thrombotic events	Relevant data for thrombotic disease*	Plasminogen activity (%)†
A	Proband	49	49	F	DVT	Negative	24
	Son	22	—	M	No	Negative	67
	Son	13	—	M	No	Negative	58
B	Proband	46	25	F	DVT	Negative	47
	Mother	74	—	F	TIA	Positive APLA	67
	Daughter	25	—	F	No	Negative	50
	Sibling	51	—	F	No	Negative	58
	Sibling	44	—	F	No	Negative	82
C	Proband	43	43	M	DVT	Negative	68
	Sibling	31	—	F	No	Negative	64
	Mother	63	—	F	No	Negative	64
	Maternal aunt	60	—	F	No	Negative	101
D	Proband	50	48	F	IS	Positive APLA	55

F: female; M: male; DVT: deep venous thrombosis; TIA: transient ischemic attack; IS: ischemic stroke; APLA: antiphospholipid antibodies.

^*^Thrombophilia investigations included lupus anticoagulant, antiphospholipid antibodies of immunoglobulin G (IgG) and IgM types, homocysteine, antithrombin, Protein C measured with two functional tests and Protein S (free) measurements.

^†^Normal range of plasminogen activity: 72% to 127%.

**Table 2:** Primers used for LR-PCR amplification of *PLG.*

PCR	PCR forward primer	Forward primer position*	PCR reverse primer	Reverse primer position*	Size (bp)
PLG_LR1	GCGCCAGCACAGAGCTCTGCTCAAC	chr6:161,121,602- 161,121,626	GCTTGCTACTTGTAAGAACTAATAACTTC	chr6:161,129,932-161,129,960	8,359
PLG_LR2	CAATTTAGCTCTCCAAACATTCTGCATCC	chr6:161,129,653-161,129,681	GAACTCCTTGAACTCACTCAAGCAATCC	chr6:161,137,405-161,137,432	7,780
PLG_LR3	AGGTACTAGATGAGTATCTTTAGGCAGG	chr6:161,137,170-161,137,197	GGCATCTCCATTGAGCTCACTGTTCC	chr6:161,144,785-161,144,810	7,641
PLG_LR4	GTCTTGCTGAACTGAGGAAGGAGACTGG	chr6:161,144,556-161,144,583	GTAGATGTCCAGCCACAGATTTCTTACC	chr6:161,152,264-161,152,291	7,736
PLG_LR5	CTGAATATTCTCCCACCTCTTGTGACC	chr6:161,152,034-161,152,060	GAACAGCTCTGTTCTGCAGTTTATTCAGG	chr6:161,159,857-161,159,885	7,852
PLG_LR6	CCCACTGCTTGGAGAAGTATGTTTAGG	chr6:161,159,629-161,159,655	TCAAGATCCAGGAGGATGGCAAAATCC	chr6:161,167,598-161,167,624	7,996
PLG_LR7	CATTTCTGAGATTCTTTCCTCAGCTTGG	chr6:161,167,413-161,167,440	TCAAAATGGGGAACAACATTTGTGTAAGG	chr6:161,175,204-161,175,232	7,820

^*^Primer positions referred to GRCh37/hg19, UCSC Genome Browser assembly, February 2009 release (http://genome.ucsc.edu/)^2^.

**Table 3:** Molecular data of the four families with plasminogen deficiency.

Family	Relationship to proband	Plasminogen activity (%)*	*PLG* mutations					Major mutations involved in thrombotic risk†
			Exon	Nucleotide change	Amino acid change	Genotype	MAF (%)	
A	Proband	24	2	c.112 A>G	p.Lys38Glu	Compound het in trans	0.27	—
			18	c.2134 G>A	p.Gly712Arg		0	—
	Son	67	2	c.112 A>G	p.Lys38Glu	Het	0.27	—
	Son	58	18	c.2134 G>A	p.Gly712Arg	Het	0	—
B	Proband	47	2	c.112 A>G	p.Lys38Glu	Het	0.27	—
	Mother	67	2	c.112 A>G	p.Lys38Glu	Het	0.27	—
	Daughter	50	2	c.112 A>G	p.Lys38Glu	Het	0.27	—
	Sibling	58	2	c.112 A>G	p.Lys38Glu	Het	0.27	—
	Sibling	82	Non-carrier	Non-carrier	Non-carrier	Non-carrier		—
C	Proband	68	7	c.781 C>T	p.Arg261Cys	Het	0	—
	Sibling	64	7	c.781 C>T	p.Arg261Cys	Het	0	—
	Mother	64	7	c.781 C>T	p.Arg261Cys	Het	0	—
	Maternal aunt	101	Non-carrier	Non-carrier	Non-carrier	Non-carrier	0	—
D	Proband	55	1	c.12 G>A	p.Lys4Lys	Compound het	0	*F12* (c.-4T>T)
			—	c.1878-6 T>C	—		0.14	*F13* (c.103 G>G)

MAF: minor allele frequency from all populations of 1000 genomes data (April 2012 v.3; www.1000genomes.org)^16^; Het:: heterozygous.

^*^Normal range of plasminogen activity: 72% to 127%.

^†^Gene (nucleotide change).

**Table 4:** *In silico* predictions of the *PLG* identified mutations.

Nucleotide Change	Protein Change	Variant Type	dbSNP v137	NNSPLICE score*	HSF score*	SIFT score (median)	PolyPhen-2 score (sensitivity - specificity)	Align GVGD score	Mutation Taster p-value	PhyloP	Grantham distances
c.12 G>A	p.Lys4Lys	PSSM	rs4252061	1.00–1.00	97.66–97.66	—	—	—	—	0.69	—
c.112 A>G	p.Lys38Glu	Missense	rs73015965	—	—	0.005 (3.37)	0.879 (0.82–0.94)	C55 (GV:0–GD:56.87)	0†	1.78	56
c.781 C>T	p.Arg261Cys	Missense	—	—	—	0 (3.84)	0.999 (0.14–0.99)	C65 (GV:0–GD:179.53)	1	4	180
c.1878-6 T>C	—	PSSM	rs192519670	0.98–0.98	84.28–84.93	—	—	—	—	−0.134	—
c.2134 G>A	p.Gly712Arg	Missense	rs202074006	—	—	0.03 (3.84)	1 (0.00–1.00)	C65 (GV:0–GD:125.13)	0.99	1.25	125

NNSPLICE [0-1]: threshold ≥0.4 and HSF [0-100]: Human Splicing Finder, threshold ≥65. In both programs a splice site effect was considered as potentially deleterious when a variation between the native and the mutation score was more than 10%; SIFT [1-0]: scores less than 0.05 indicate substitutions are predicted as deleterious; Polyphen-2 [0-1]: scores range from 0.000 (most probably benign) to 1 (most probably damaging); Align GDGV [C0-C65]: scores range from Class C0 (less likely deleterious) to Class C65 (most likely deleterious); Mutation Taster [0-1]: from disease causing variants (p-value = 1.0) to might not be disease causing (p-value <0.99); PhyloP [-14.1 to 6.4]: from highly conserved (score >3) to moderately conserved (score 1-3) or poorly conserved (score <1); Grantham distances [0-215]: from highly different physicochemical properties and more probably damaging (score >50) to moderately (score 25-50) or poorly different and most probably tolerated (score <25); PSSM: Potential splicing site mutation.

^*^Score native – mutation.

^†^This variant is listed as pathogenic in NCBI ClinVar database (http://www.ncbi.nlm.nih.gov/clinvar/). Thus, it is automatically predicted to be disease-causing in Mutation Taster but real probability is shown.
